# Sex/gender differences in individual and joint trajectories of common mental health symptoms in early to middle adolescence

**DOI:** 10.1002/jcv2.12057

**Published:** 2021-12-11

**Authors:** Aja Louise Murray, Anastasia Ushakova, Lydia Speyer, Ruth Brown, Bonnie Auyeung, Xinxin Zhu

**Affiliations:** ^1^ Department of Psychology University of Edinburgh Edinburgh UK; ^2^ Center for Health Informatics, Computing and Statistics Medical School Lancaster University Lancaster UK; ^3^ Department of Psychology University of Cambridge Cambridge UK

**Keywords:** ADHD, adolescence, conduct problems, internalizing problems, latent class growth analysis, mental health, trajectories

## Abstract

**Background:**

Early to middle adolescence is a critical period of development for mental health issues. Illuminating sex/gender differences in mental health trajectories in this period is important for targeting screening and preventive interventions; however, evidence to date on the extent and nature of sex/gender differences in common mental health issue trajectories in this period has produced mixed findings. There is a particular gap in our knowledge of sex/gender differences in the joint trajectories of commonly co‐occurring mental health issues in adolescence, given the strong tendency for mental health issues to co‐occur.

**Method:**

We applied sex/gender‐stratified latent class growth analysis to an age‐heterogeneous cohort (age 10–15) derived from the population‐representative *UK Household Longitudinal Study*. We explored sex/gender differences in attention deficit hyperactivity disorder (ADHD) symptoms, internalizing problems, and conduct problems individually and jointly.

**Results:**

We found indications of sex/gender differences in a number of areas. There were fewer classes in the optimal model to describe the heterogeneity in internalizing problems and conduct problems trajectories in males and females respectively. Further, for ADHD, affected males were more likely to enter adolescence with already elevated symptoms whereas affected female trajectories were characterized by an escalation of symptoms during adolescence.

**Conclusions:**

There are sex/gender differences in the levels and trajectories of specific mental health symptoms in early to middle adolescence; however, in both males and females there is a strong tendency for multiple issues to co‐occur.

**Key points**

- Early to middle adolescence is a critical period for mental health, sex/gender differences in mental health are prominent, and mental health issues tend to co‐occur.
- Attention deficit hyperactivity disorder symptoms appear to have a later onset for females than for males, with conduct problems being a more prominent co‐occurring issue for males and internalizing problems for females.
- Preventive interventions should be transdiagnostic, reflecting the tendency for issues to co‐occur and gender/sex‐tailored interventions, reflecting that males and females tend to have different profiles of mental health issues in early to middle adolescence, should be explored.

## INTRODUCTION

Early to middle adolescence is a critical period of development where mental health issues are particularly liable to emerge or escalate (Kessler et al., 2005). Alongside this developmental change in mental health issue risk, gender and sex differences are among the most well‐established findings in mental health research. “Sex” refers to biological/physiological characteristics that define, for example, males versus females while “gender” refers to socially constructed roles, behaviors, activities, and attributes that society considers appropriate for, for example, males versus females. However, male and female are only two possible gender identities, defined as the gender that a person identifies with (including no gender). It can be difficult to separate the effects of sex and gender because gendered socialization tends to begin early in life. For this reason, unless otherwise stated, we use the term “sex/gender” to acknowledge their overlap. Thus defined, those of female sex/gender tend to exhibit higher levels of internalizing problems such as anxiety and depression and males tend to exhibit higher levels of externalizing problems such as conduct and oppositional defiant disorder, as well as and neurodevelopmental issues including attention deficit hyperactivity disorder (ADHD) symptoms (see e.g., Booth & Murray, 2018, for a review). There is also emerging evidence that males and females differ in their developmental trajectories of mental health symptoms, including in adolescence (Diamantopoulou et al., 2011; Lewis et al., 2020; Murray et al., 2018; Nelemans et al., 2014; Ohannessian et al., 2017). However, the extent and nature of sex/gender differences in mental health trajectories in adolescence remains poorly understood.

Given that averaged developmental trajectories of mental health symptoms have been shown to conceal considerable heterogeneity, trajectory analysis methods such as latent class growth analysis are arguably particularly valuable for illuminating sex/gender differences in the development of mental health symptoms (Nagin & Odgers, 2010; Ohannessian et al., 2017). These methods help to organize individual differences in developmental trajectories into a small number of more manageable and potentially clinically useful developmental classes. Sex/gender differences can then be examined via multi‐group models (Diamantopoulou et al., 2011), sex/gender‐stratified analyses (Murray, Zhu, et al., 2021), or by predicting developmental trajectory class membership from sex/gender (Murray et al., 2020; Ohannessian et al., 2017).

Differences in designs (e.g., measurement points, sample characteristics, and mental health measures) make it difficult to directly compare findings from different trajectory analysis studies and findings to date are mixed. In particular, it is unclear the extent to which there are important sex/gender trajectory differences that reflect more than merely a greater risk in a particular sex/gender across *all* stages of development (Diamantopoulou et al., 2011; Kwong et al., 2019; Lewis et al., 2020). For example, using data from the Avon Longitudinal Study of Parents and Children (ALSPAC) cohort, Kwong et al. (2019) found that females were more likely to belong to all four subgroups characterized by an elevation of internalizing problems between childhood and adulthood (“childhood persistent”, “early adulthood onset”, “childhood limited”, and “adolescent limited”) compared to an unaffected (“stable low”) group. Similarly, Ohannessian et al. (2017) found that females were more likely to belong to all adolescent generalized anxiety classes characterized by higher levels of symptoms (“very high decreasing rapidly”, “high decreasing”, and “moderate decreasing slightly”). On the other hand, for social anxiety, females were more also more likely to be in the “low stable” in comparison to the “low increasing” class, suggesting that while females may enter adolescence with higher levels of symptoms, males may be more likely to show an increase over this period.

Studies that have taken a multi‐group or sex/gender‐stratified analysis approach have, however, provided hints that the shapes of internalizing developmental trajectories around this critical period may differ for males and females within analogous trajectory groups. For example, examining developmental trajectories of depression across adolescence (ages 11–18), Diamantopoulou et al. (2011) found three trajectory groups: “low”, “increasing,” and “decreasing.” Among those with increasing symptom trajectories, the increase was faster for females and among those with decreasing trajectories, the decrease was faster for males, suggesting a quicker onset and slower recovery/greater persistence for females.

For mental health issues characterized by an over‐representation of males the picture is similarly mixed. Some studies have found sex/gender differences only in overall levels of but not the trajectory shapes of ADHD symptoms and conduct problems across periods that include adolescence (Diamantopoulou et al., 2011; Döpfner et al., 2015). However, one recent study using sex/gender‐stratified latent class growth analysis found that the subgroup of females who showed an escalation of conduct problems in adolescence tended to show this peak later than the corresponding male subgroup (Murray, Zhu, et al., 2021). Similarly, some studies have suggested that females who show elevated ADHD symptoms are more likely than males to belong to a trajectory group with an onset around adolescence compared to earlier (Murray et al., 2018; Murray, Hall, et al., 2021). Further studies are needed to clarify the nature and extent of sex/gender differences in internalizing problems, externalizing problems, and ADHD in adolescence.

Additional complexity in understanding sex/gender differences in adolescent mental health trajectories derives from the strong tendency of symptoms in different domains to co‐occur (Murray et al., 2016) but not necessarily in the same way for males and females. For example, there is evidence that females and males with ADHD show relatively higher levels of co‐occurring internalizing and externalizing problems respectively (Williamson & Johnston, 2015). Given that secondary symptoms may interact with and/or modify primary symptoms (Drabick & Kendall, 2010), examination of sex/gender differences in joint trajectories of symptoms is important. There have, however, only been a handful of studies examining sex/gender differences in co‐occurring mental health issue trajectories covering the adolescent period (Chen & Simons‐Morton, 2009; Diamantopoulou et al., 2011; Patalay et al., 2017; Speyer et al., 2021). One study examined joint developmental trajectories of ADHD, internalizing, and externalizing symptoms across ages 7–15 (Murray et al., 2020). Using the “unaffected” class as the reference class, they found that male sex/gender increased the likelihood of membership in several classes characterized by elevations of symptoms in all three domains but not in a class characterized only by elevations in internalizing problems. Speyer et al. (2021) found that male sex increased the likelihood of membership in age 4–16 trajectory classes characterized by high internalizing and externalizing symptoms; moderate externalizing symptoms; and high externalizing symptoms compared to the unaffected class. However, male sex did not increase the likelihood of membership in their class characterized by moderate internalizing and externalizing levels. Taken together, these findings suggest that males may be more liable to show trajectories characterized by multiple co‐occurring issues that include externalizing problems.

While there are indications for sex/gender differences in trajectories of mental health issues, there remains a lack of clarity on their existence and nature in adolescence, with a particular gap in our knowledge of joint developmental trajectories of multiple co‐occurring issues. Improved knowledge of mental health trajectories within this critical period can inform optimal timing of screening and preventive efforts. For example, if females tend to show later onset of symptoms in a particular domain then such efforts may be better targeted to females at a later stage. Similarly, it can help ensure that frontline professionals are attuned to symptom onsets at appropriate stages for all sexes/genders, especially for sex/gender‐atypical symptoms (e.g., ADHD for females or internalizing problems for males) that may already be more liable to be missed (Williamson & Johnston, 2015). Further, diagnostic criteria for certain disorders currently employ age cut‐off criteria, either to quality for a diagnosis (e.g., the age 12 symptom onset cut‐off for ADHD) or for specifiers (e.g., the age 10 cut‐off for early vs. late onset subtypes for conduct disorder) (American Psychiatric Association, 2013). These currently assume that a single cut‐off is appropriate, irrespective of sex/gender. Evidence that males and females differ in their developmental trajectories in adolescence could, however, imply that sex/gender‐adjusted criteria could provide greater clinical utility in detecting and classifying affected adolescents. Finally, improved knowledge of sex/gender differences in joint developmental trajectories of symptoms can help predict which additional symptoms an adolescent may be at concurrent or later risk of when presenting with symptoms in a particular domain (e.g., female ADHD symptoms may predict later internalizing symptoms but male ADHD symptoms may indicate conduct problems risk). The goal of the current study is, therefore, to use an age‐heterogeneous cohort design to examine sex/gender differences in individual and joint trajectories of ADHD symptoms, conduct problems, and internalizing problems as three of the most common and significant mental health/neurodevelopmental issues experienced in adolescence.

## METHOD

### Participants

Data came from the UK Household Longitudinal Study (UKHLS), a UK longitudinal survey of approximately 40,000 baseline households, beginning in 2009. Using these data, an adolescent development cohort was constructed using the participants who were aged 10, 11, and 12, in the wave 1 data collection (2009–2010) for UKHLS. This provided an age‐heterogeneous cohort design (also known as an accelerated cohort design) where multiple age cohorts were followed across three measurement points. Accelerated cohort designs involve tracking development from multiple starting ages at once to maximize the age coverage of a study and facilitate longitudinal modeling of a given developmental period without having to wait for participants to age through this whole period. Indeed, different age cohorts (e.g., with starting ages of 10, 11, and 12) can be combined in the same analysis as if participants were all from the same age cohort but with missing data points specified for the ages where data had not (yet) been collected for a given participant. However, the disadvantage of this is that it assumes that the developmental processes being modeled are identical across the cohorts. This is the “invariance” assumption.

Participants provided self‐reported mental health symptom data at waves 1, 3, and 5, providing coverage of ages 10, 11, 12, 13, 14, and 15. In total, data from *n* = 4866 participants were utilized; *n* = 2453 females and *n* = 2415 males. Participants were organized into age cohorts of one‐year age band length to attempt to strike an optimal balance between minimizing missingness and maximizing the temporal (age) resolution of the data. The coverage of each age is provided in Table S1, along with descriptive statistics for each mental health domain. Missing data were dealt with using full information maximum likelihood estimation in combination with the attrition weights provided as part of the UKHLS data release.

### Measures

#### ADHD, conduct problem and internalizing problem symptoms

ADHD, conduct problem, and internalizing symptoms were measured using the self‐report version of the *Strengths and Difficulties Questionnaire* (*SDQ*; Goodman, 1997). Five items measure each symptom domain, each on a 3‐point scale with response options: not true; somewhat true; and certainly true. Composite scores are derived by summation of individual item responses to give a possible score range of 0–10. Composite scores were not created/utilized for adolescents with more than two item scores missing. For ADHD, previous research has suggested that scores above 6 represent borderline scores while scores above 7 represent clinically significant scores (Caye et al., 2016; Riglin et al., 2016). For the other domains, clear clinical cut‐offs have not been established. The psychometric properties of the SDQ has been extensively evaluated in previous studies (see e.g., Kersten et al., 2016, for a review). While there have been some questions raised over its optimal factor structure and internal consistency, these studies have generally supported its use as a measure of common mental health in adolescence. Importantly, previous studies have supported its sex/gender and developmental invariance over similar developmental periods to that covered in the present study (Murray, Speyer, et al., 2021).

### Statistical procedure

For ADHD symptoms, conduct problems, and internalizing problems individually and then jointly, sex/gender‐stratified latent class growth analysis models were fit with increasing numbers of classes until a stopping point defined by a non‐significant Lo‐Mendell‐Rubin (LMR) likelihood ratio test. A non‐significant adjusted LMR test indicates that a *k* class model is not significantly better fitting than the corresponding *k*−1 class. AIC, BIC, saBIC and entropy are provided for additional information. Previous research has noted that there is no one best class enumeration index that will consistently select the “correct” number of classes over different contexts and as such class enumeration indices are best used to inform but not solely determine the selection of numbers of classes (Whittaker & Miller, 2021). Moreover, when conceptualizing the latent class models as a means of providing a convenient but defensible discretization of an underlying continuous distribution, there is no ‘correct’ number of classes; only an optimal number for a given purpose (Nagin et al., 2018). We, therefore, also considered the substantive meaning of classes (e.g., whether substantively meaningful distinctions are revealed in a *k* model compared to a *k*−1 class model) and practical considerations such as preserving parsimony/interpretability and avoiding very small class sizes. In discretizing a continuous distribution, selecting different numbers of classes for males versus females is also better thought of as reflecting a tendency towards requiring more classes to capture heterogeneity, as opposed to a qualitative or absolute distinction.

Models with intercept, linear slope, and quadratic slope factors were fit because previous research has suggested that mental health symptom trajectories may be non‐linear in adolescence (e.g., Murray et al., 2018). Within‐class factor variances and covariances were fixed to 0. This can be thought of as an operationalization of the assumption that the trajectory groups are convenient discretizations of a continuous distribution as opposed to interpretable as ‘true’ categories (Nagin & Odgers, 2010). This is in contrast to growth mixture modeling which has been interpreted as viewing the groups as reflecting subpopulations. It thus allows variation around an average growth curve within each subpopulation (see e.g., Nagin & Odgers, 2010, for a discussion). In the joint model, a parallel process model specification was used in which classes were defined by trajectories in all three domains at once. This can be contrasted with the approach of defining separate latent categorical variables for each of the symptom domains and then examining the associations between the domains at the level of these categorical variables. The advantage of the parallel process approach is that it allows joint trajectories to emerge that better reflect the combined trajectories of different mental health domains as these may differ from those that emerge when considering each mental health domain in isolation. All models were fit in Mplus 8.4 using robust maximum likelihood estimation (Muthén & Muthén, 2015).

## RESULTS

### Model selection

**FIGURE 1:**
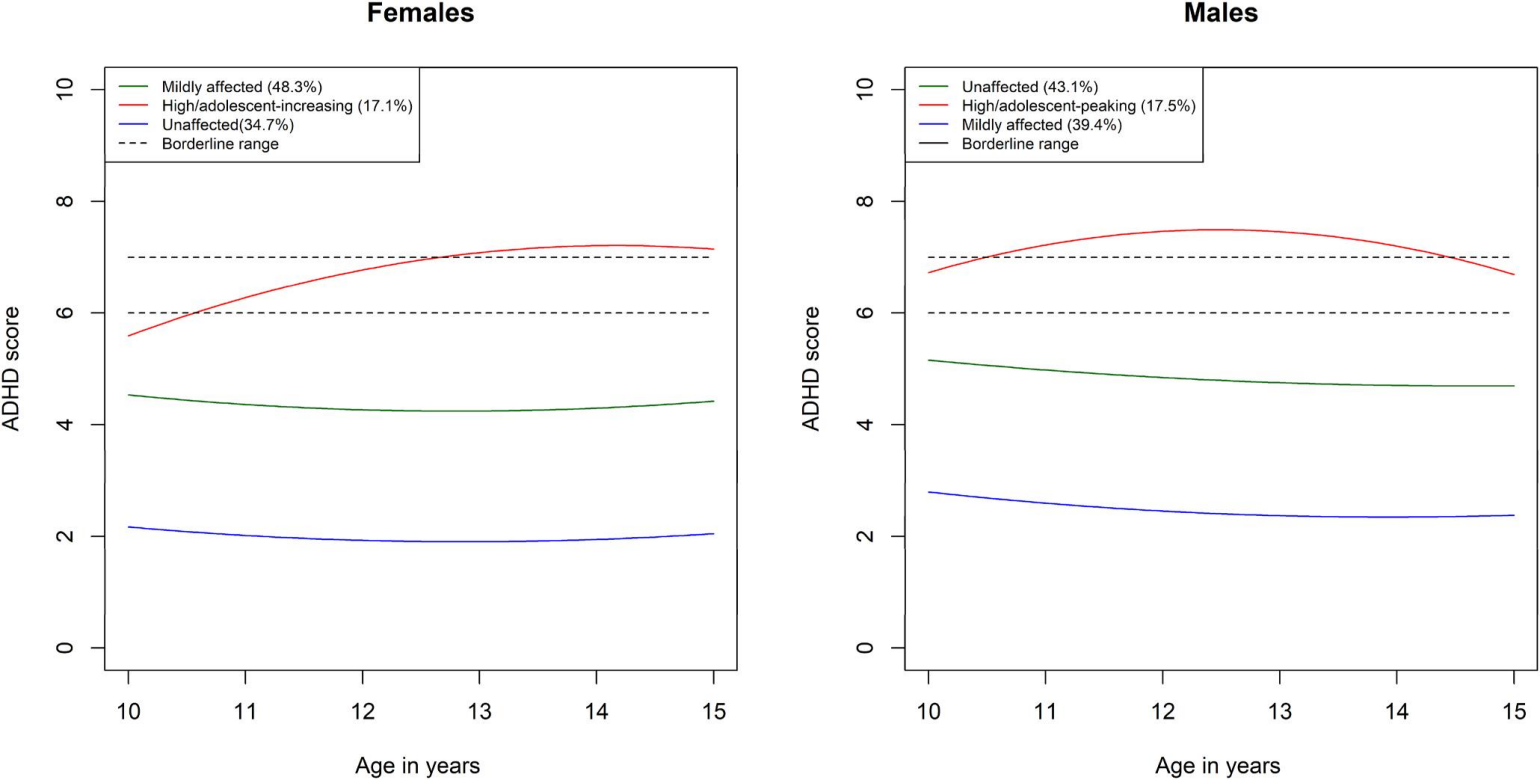
Trajectories for the three attention deficit hyperactivity disorder (ADHD) classes in the female and male subsamples

**FIGURE 2:**
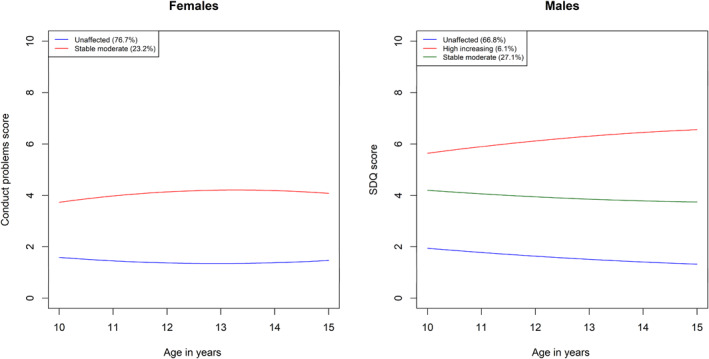
Conduct problems trajectories

**FIGURE 3:**
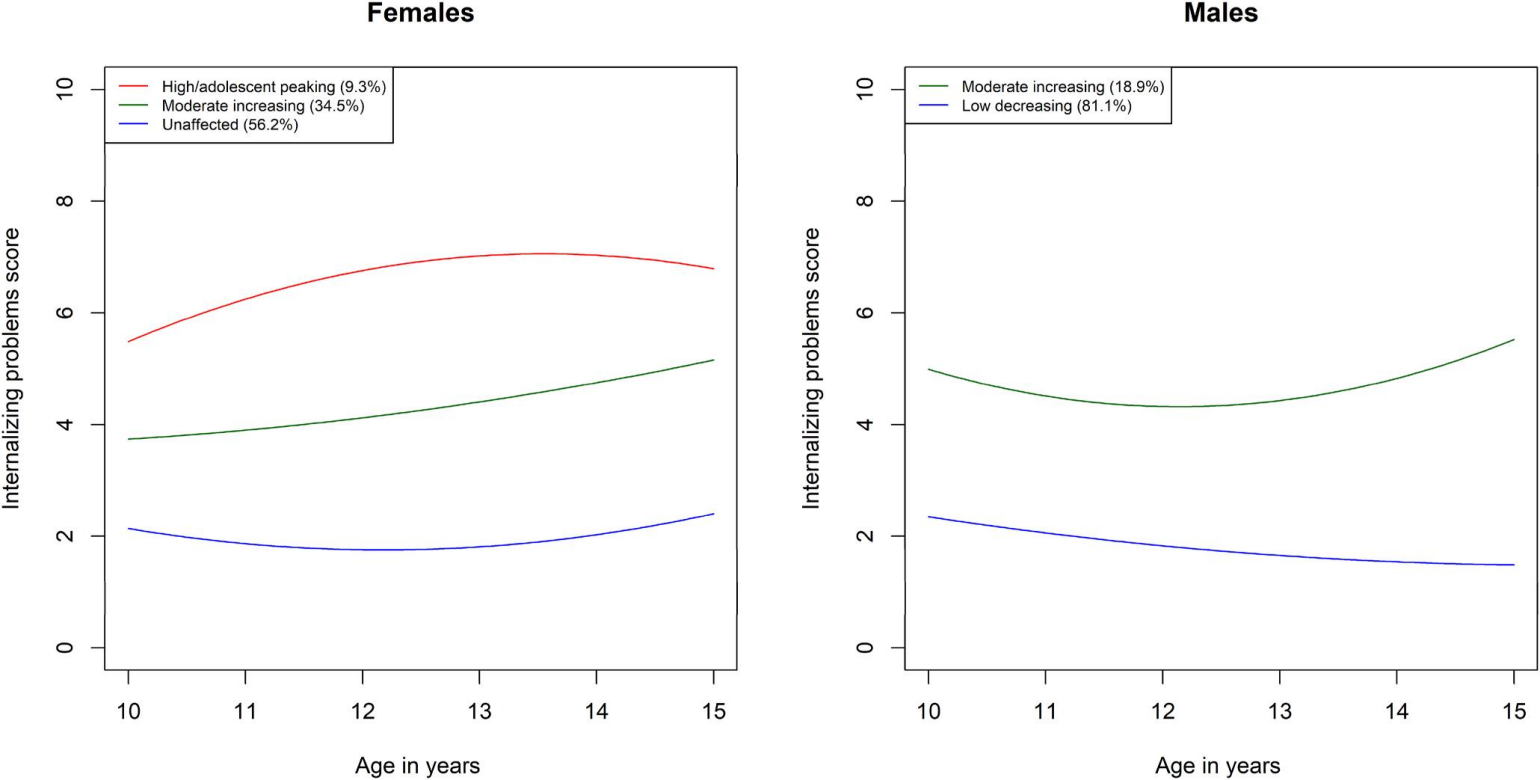
Internalizing problems trajectories

**FIGURE 4:**
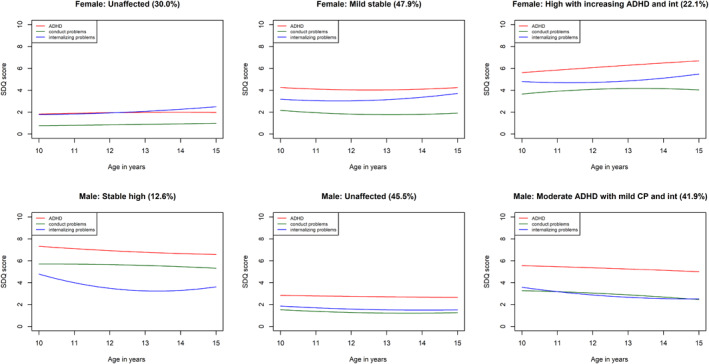
Joint attention deficit hyperactivity disorder (ADHD), conduct problems, and internalizing problems trajectories

Fit statistics for models with varying numbers of classes for the individual mental health trajectories in the female and male‐subsamples are provided in Tables S2–S5 up to the point where a non‐significant LMR test was reached. For the female subsample, the LMR test selected a 3‐class model for ADHD symptoms, a 2‐class model for conduct problems, a 3‐class model for internalizing problems, and a 3‐class models for the joint trajectory groups. For the male subsample, the LMR test selected a 3‐class model for ADHD symptoms, a 3‐class model for conduct problems, and a 2‐class model for internalizing problems. In terms of joint trajectory groups, in the male subsample, the LMR test selected a 3‐class model, while the female subsample selected a 3‐class model. These are summarized in Tables 1, 2, 3, 4 and Figures 1, 2, 3, 4. Comprehensive descriptions of the classes are provided in Appendix S1. Full model outputs (for up to 6‐class models) are provided at: https://osf.io/bv5sh/

**TABLE 1:** Parameters for the optimal ADHD trajectory models

Class	Label	Size^a^	Intercept mean	SE	Linear slope mean	SE	Quadratic slope mean	SE
Females								
1	Mildly affected	48.3%	4.534	0.217	−0.207	0.144	0.037	0.023
2	High/adolescent‐increasing	17.1%	5.593	0.405	0.775	0.261	−0.093	0.043
3	Unaffected	34.7%	2.170	0.242	−0.184	0.180	0.032	0.031
Males								
1	High/adolescent‐peaking	17.5%	6.725	0.360	0.618	0.281	−0.125	0.049
2	Mildly affected	39.4%	2.793	0.203	−0.228	0.170	0.029	0.031
3	Unaffected	43.1%	5.154	0.352	−0.197	0.198	0.021	0.035

Abbreviation: ADHD, attention deficit hyperactivity disorder.

^a^ Based on estimated posterior probabilities.

**TABLE 2:** Parameters for the optimal conduct problem trajectory models

Class	Label	Size^a^	Intercept mean	SE	Linear slope mean	SE	Quadratic slope mean	SE
Females								
1	Unaffected	76.7%	1.590	0.097	−0.164	0.074	0.028	0.015
2	Stable moderate	23.3%	3.734	0.257	0.289	0.206	−0.044	0.042
Males								
1	Unaffected	66.8%	1.942	0.106	−0.173	0.078	0.010	0.014
2	High increasing	6.1%	5.638	0.647	0.279	0.386	−0.019	0.059
3	Stable moderate	27.1%	4.200	0.268	−0.151	0.191	0.012	0.037

^a^ Based on estimated posterior probabilities.

**TABLE 3:** Parameters for the optimal internalizing problem trajectory models

Class	Label	Size^a^	Intercept mean	SE	Linear slope mean	SE	Quadratic slope mean	SE
Females								
1	High/adolescent peaking	9.3%	5.485	0.712	0.886	0.474	−0.125	0.087
2	Moderate increasing	34.5%	3.741	0.268	0.128	0.225	0.031	0.039
3	Unaffected	56.2%	2.137	0.164	−0.352	0.114	0.081	0.021
Males								
1	Moderate increasing	18.9%	4.992	0.370	−0.629	0.272	0.147	0.048
2	Low decreasing	81.1%	2.346	0.152	−0.317	0.100	0.029	0.016

^a^ Based on estimated posterior probabilities.

**TABLE 4:** Parameters for the optimal joint ADHD, conduct problem, and internalizing problem trajectory models

Class	Label	Size^a^	ADHD intercept mean (SE)	ADHD linear slope mean (SE)	ADHD quadratic slope mean (SE)	CP intercept mean (SE)	CP linear slope mean (SE)	CP quadratic slope mean (SE)	Int intercept mean (SE)	Int linear slope mean (SE)	Int quadratic slope mean (SE)
Females											
1	Unaffected	30.0%	1.825 (0.216)	0.085 (0.135)	−0.011 (0.023)	0.760 (0.111)	0.032 (0.084)	0.002 (0.015)	1.776 (0.170)	0.034 (0.124)	0.022 (0.022)
2	Mild stable	47.9%	4.259 (0.178)	−0.182 (0.124)	0.036 (0.021)	2.167 (0.133)	−0.255 (0.084)	0.041 (0.015)	3.181 (0.187)	−0.195 (0.124)	0.060 (0.021)
3	High with increasing ADHD and int	22.1%	5.614 (0.243)	0.235 (0.177)	−0.004 (0.031)	3.649 (0.217)	0.313 (0.161)	−0.047 (0.030)	4.799 (0.270)	−0.158 (0.219)	0.059 (0.039)
Males											
1	Stable high	12.6%	7.320 (0.439)	−0.234 (0.338)	0.017 (0.058)	5.696 (0.368)	0.010 (0.294)	−0.017 (0.052)	4.781 (0.503)	−0.928 (0.404)	0.139 (0.077)
2	Unaffected	45.5%	2.837 (0.167)	−0.052 (0.116)	0.003 (0.021)	1.523 (0.129)	−0.174 (0.081)	0.024 (0.014)	1.859 (0.152)	−0.186 (0.103)	0.023 (0.017)
3	Moderate ADHD symptoms with mild CP and int	41.9%	5.557 (0.223)	−0.088 (0.159)	−0.005 (0.029)	3.268 (0.176)	−0.073 (0.110)	−0.018 (0.021)	3.577 (0.208)	−0.442 (0.153)	0.046 (0.030)

Abbreviations: ADHD, attention deficit hyperactivity disorder; CP, conduct problems; Int, internalizing problems.

^a^ Based on estimated posterior probabilities.

## DISCUSSION

The purpose of the present study was to illuminate sex/gender differences in the early to middle adolescence trajectories of ADHD symptoms, conduct problems, and internalizing problems. We found several sex/gender differences in the individual and joint developmental trajectories of these symptom domains, both in terms of a tendency towards different numbers of classes in the optimal models and in terms of the trajectories within these classes. For both internalizing and conduct problems, different numbers of classes were judged optimal to model heterogeneity in male versus female trajectories. Specifically, a more severely affected group for conduct problems and internalizing problems was absent for females and males respectively. For ADHD symptoms, heterogeneity was optimally characterized by three classes for both males and females; however, the affected class showed subtle but important sex/gender differences. In particular, males were more likely to enter adolescence with symptoms in the borderline range and these quickly escalated into the clinical range. Females, however, were more likely to begin with symptoms outside the borderline range and show an escalation later in adolescence, increasing into the clinical range only around age 13. In our analysis of joint trajectories, three classes were judged optimal to characterize heterogeneity in both males and females and in both cases the severity of symptoms across all three domains tended to track each other across classes. The primary sex/gender difference was that ADHD symptoms and conduct problems were generally higher for males.

The lack of a more severely affected conduct problems trajectory in the model judged optimal for females suggests that such a trajectory is not sufficiently common to be detected in our sample. It is consistent with other recent research that has suggested that the escalation of antisocial behavior in adolescence observed at the aggregate level is driven primarily by a small subset of primarily male youth (Murray, Zhu, et al., 2021). This group, representing 6.1% of males in the current study, showed elevated symptoms on entry to adolescence with a subsequent steady increase up to age 15. The remaining two groups were similar across males and females and were characterized by low or moderate but stable symptoms across adolescence. This suggests that prevention efforts would be best targeted at the small subgroup of male youth with high and escalating symptoms. Though sex/gender differences in conduct problems are widely known, the reasons underlying their greater tendency to escalate in males in adolescence are not. A recent review (Murray, Mirman, et al., 2021), for example, highlighted that sex/gender differences have been largely neglected in dominant adolescent risk‐raking models of conduct problems and are only now beginning to gain explicit attention. Further illumination of the drivers of sex/gender differences can help with optimization of prevention efforts.

For internalizing problems, it was males who showed a lack of more severely affected trajectory category in the model judged optimal for this sample, indicating that such a trajectory was not sufficiently common to be detected in the current sample. Females characterized as more severely affected represented 9.2% of the female subsample and showed trajectories with initially elevated symptoms on entry to adolescence but with further increases over the course of early to middle adolescence. Their trajectory was curvilinear and symptoms appeared to be peak around age 13–14, consistent with the peak age of onset for disorders such as depression and social anxiety disorder (Kessler et al., 2005). The presence of this group only in the model judged optimal for females is consistent with previous evidence that suggests that pubertal hormone levels are generally more closely linked to internalizing problems in females than in males; that females may be more sensitive to the effects of social changes (e.g., greater sensitivity to peer rejection) and to experience self‐concept issues precipitated by the identity developmental changes occurring in adolescence (see e.g., Rapee et al., 2019, for a review). Taken together, these two sets of findings suggest that it will be worthwhile exploring whether males and females benefit from tailored preventive interventions to prepare them for adolescence, respectively with more emphasis on behavioral versus emotional problems.

Though the same number of classes were judged optimal to characterize male and female heterogeneity in ADHD symptoms, there were sex/gender differences in the more severely affected class. Males showed a trajectory characterized by already borderline symptom levels on entry to adolescence (increasing into the clinical range shortly thereafter) while the analogous female class showed symptoms that only increased into the borderline then clinical range later. This is consistent with growing evidence that among youth affected by ADHD symptoms, females may be more likely to show an onset of supra‐threshold symptoms only after adolescence (Murray et al., 2018; Murray, Hall, et al., 2021). Given that ADHD is still often viewed as a disorder of childhood (and indeed age of onset is by diagnostic criteria before age 12) this difference in age of onset may contribute to the under‐identification of females with ADHD (Williamson & Johnston, 2015). As such, professionals should be aware that ADHD symptoms may be more liable to show clinically significant levels later in females and thus be attuned to symptoms at later developmental stages. Further, the findings raise questions about whether the age 12 cut‐off is too strict and may particularly disadvantage females. Nevertheless, the prevalence and escalation of symptoms of adolescence underlines the importance of the adolescent period for these symptoms and the importance of continuing to provide adequate support. Increases could reflect a range of factors, such as the impact of new stressors, increasing expectations of independence, and normative escalations in sensation‐seeking compounding pre‐existing self‐regulation difficulties and/or overwhelming previously compensating factors such as high IQ or parental support (Agnew‐Blais et al., 2016; Murray, Hall, et al., 2021; Vos et al., 2021).

Finally, our analyses of the joint trajectories of ADHD, conduct problems, and internalizing problems highlighted that mental health symptoms tend to cluster together and track each other developmentally in early to middle adolescence. This is consistent with previous observations of their high levels of co‐occurrence in this period (Murray et al., 2016) and suggests that the most promising prevention efforts will be transdiagnostic in nature and/or be based on evidence regarding possible (bi‐) directional influences between different domains. However, there were also sex/gender differences in joint trajectories with the most severely affected group in females showing less severe ADHD and conduct problem symptoms and more severe internalizing problem symptoms. This, again points to the potential benefits to tailoring prevention to sex/gender based on the most prevalent symptoms experienced in males versus females.

### Limitations

A primary limitation is that we employed a mental health measure in which each domain is captured by only a small number of items. As such, we could not make distinctions between different sub‐domains of ADHD, conduct problems, and internalizing problem symptoms and these sub‐domains may show different gender differences in trajectories (Murray et al., 2018). Similarly, while optimal clinical cut‐offs have been estimated for total scores and for ADHD; there are not reliable cut‐off scores for the other subscales of the measure and even the total score and ADHD cut‐offs are debated (e.g., Wolpert et al., 2015). We also relied on a single informant, whereas multi‐informant approaches can better capture adolescent mental health across different contexts and in interaction with different informants. Second, while using an age‐heterogeneous design allowed us to capture the early to middle adolescent period with relatively high temporal resolution, such designs rely on the assumption of invariance in trajectories across the age cohorts; an assumption that would be violated if adolescents born in different years showed differences in their mental health development. Further, even with using one year age bands we still had high levels of missingness for each measurement point. This may have contributed to the low entropy values for some of the class solutions. Low entropy was particularly an issue for the ADHD optimal class solutions. A possible reason for this is the difficulty of measuring ADHD symptoms in adolescence and, in particular, differentiating them from normative increases in sensation‐ and reward‐seeking behaviors that occur around this time (Shulman et al., 2016). Using a more comprehensive measure of ADHD symptoms and making distinctions between the inattention, hyperactivity, and impulsivity domains would likely have helped achieve better separation between the classes. Third, we were only able to address male versus female differences as we did not have sufficient participants with other gender identities to meaningfully examine these. Finally, there are numerous subjective aspects of latent class growth analysis, including class enumeration. As such, different class solutions could be judged optimal by different researchers based on different theoretical stances and weighing of factors such as parsimony.

## CONCLUSIONS

There was evidence for sex/gender differences in developmental trajectories of symptoms of common and commonly co‐occurring mental health issues in the critical period of early‐to‐middle adolescence suggesting that exploration of tailoring of prevention efforts to sex/gender is likely to be beneficial. Aside from conduct problems being more prominent in males and internalizing problems in females, findings suggest that females may be more likely to show later onsets of clinically significant ADHD symptoms. ADHD symptoms, conduct problems, and internalizing problems tended to co‐occur for males and females, suggesting that transdiagnostic approaches to prevention are likely to be most widely efficacious for reducing the mental health problems in adolescence.

## CONFLICT OF INTEREST

The authors have declared that they have no competing or potential conflict of interests.

## ETHICS STATEMENT

The data used in the current study was from the Understanding Society study, which received ethical approval from the University of Essex Ethics Committee.

## Supporting information

Supporting Information S1

## Data Availability

Data are available from: https://beta.ukdataservice.ac.uk/datacatalogue/series/series?id=2000053.
