# Supplementary material for: Sex/gender differences in individual and joint trajectories of common mental health symptoms in early to middle adolescence
Source: JCPP Adv. 2021 Dec 11;2(1):e12057. doi: 10.1002/jcv2.12057 (PMC10242831; doi:10.1002/jcv2.12057)
Supplement: Supplementary file 1 — Supporting Information S1 [file JCV2-2-e12057-s001.docx]

**Table S1: Descriptive Statistics**

|  | **Females** | | | **Males** | | |
| --- | --- | --- | --- | --- | --- | --- |
| **Variable** | **N** | **Mean** | **SD** | **N** | **Mean** | **SD** |
| ADHD age 10 | 391 | 4.017 | 2.278 | 399 | 4.412 | 2.474 |
| ADHD age 11 | 433 | 3.546 | 2.182 | 399 | 4.485 | 2.489 |
| ADHD age 12 | 605 | 3.912 | 2.324 | 657 | 4.367 | 2.376 |
| ADHD age 13 | 676 | 3.921 | 2.239 | 658 | 4.206 | 2.302 |
| ADHD age 14 | 815 | 4.042 | 2.383 | 795 | 4.167 | 2.365 |
| ADHD age 15 | 876 | 3.989 | 2.282 | 798 | 4.154 | 2.227 |
| Conduct problems age 10 | 391 | 2.179 | 1.737 | 399 | 2.693 | 1.911 |
| Conduct problems age 11 | 433 | 1.948 | 1.626 | 399 | 2.693 | 1.957 |
| Conduct problems age 12 | 604 | 2.019 | 1.731 | 657 | 2.460 | 2.004 |
| Conduct problems age 13 | 677 | 2.007 | 1.671 | 658 | 2.493 | 1.870 |
| Conduct problems age 14 | 815 | 2.126 | 1.788 | 794 | 2.347 | 1.837 |
| Conduct problems age 15 | 876 | 2.011 | 1.621 | 798 | 2.276 | 1.817 |
| Internalizing problems age 10 | 391 | 3.329 | 2.193 | 399 | 2.859 | 2.263 |
| Internalizing problems age 11 | 433 | 2.796 | 2.103 | 398 | 2.670 | 2.178 |
| Internalizing problems age 12 | 605 | 3.084 | 2.196 | 657 | 2.315 | 1.924 |
| Internalizing problems age 13 | 677 | 3.161 | 2.192 | 658 | 2.244 | 1.979 |
| Internalizing problems age 14 | 815 | 3.609 | 2.363 | 795 | 2.110 | 1.811 |
| Internalizing problems age 15 | 876 | 3.604 | 2.249 | 798 | 2.221 | 2.031 |

**Table S2: Model fits for female and male subsamples for ADHD symptoms**

| **N Classes** | **LMR** | ***p*** | **AIC** | **BIC** | **saBIC** | **Entropy** |
| --- | --- | --- | --- | --- | --- | --- |
| **Female subsample** | | | | | |  |
| **1** |  |  | 17295.000 | 17347.246 | 17318.651 | - |
| **2** | 403.615 | <.001 | 16886.457 | 16961.923 | 16920.619 | .496 |
| **3** | **125.387** | **<.001** | **16765.055** | **16863.741** | **16809.728** | **.532** |
| **4** | 24.687 | .181 | 16747.577 | 16869.484 | 16802.762 | .447 |
| **Male subsample** | | | | | | |
| **1** |  |  | 16822.604 | 16874.709 | 16846.114 | - |
| **2** | 427.257 | <.001 | 16389.634 | 16464.897 | 16423.593 | .520 |
| **3** | **65.028** | **.003** | **16330.519** | **16428.940** | **16374.927** | **.484** |
| **4** | 28.130 | .166 | 16309.486 | 16431.065 | 16364.343 | .517 |

*Note.* Model selected based on LMR test shown in bold.

**Table S3: Model fits for female and male subsamples for conduct problems**

| **N Classes** | **LMR** | ***p*** | **AIC** | **BIC** | **saBIC** | **Entropy** |
| --- | --- | --- | --- | --- | --- | --- |
| **Female subsample** | | | | | |  |
| **1** | - | - | 15016.016 | 15068.265 | 15039.670 | - |
| **2** | **611.150** | **<.001** | **14393.292** | **14468.763** | **14427.459** | **.690** |
| **3** | 209.558 | .1154 | 14185.022 | 14283.715 | 14229.702 | .740 |
| **Male subsample** | | | | | |  |
| **1** | - | - | 15214.075 | 15266.176 | 15237.581 | - |
| **2** | 611.788 | <.001 | 14590.651 | 14665.908 | 14624.604 | .729 |
| **3** | **132.905** | **.0026** | **14461.480** | **14559.894** | **14505.881** | **.713** |
| **4** | 84.435 | .4598 | 14382.335 | 14503.905 | 14437.183 | .637 |

*Note.* Model selected based on LMR test shown in bold.

**Table S4: Model fits for female and male subsamples for internalizing problems**

| **N Classes** | **LMR** | ***p*** | **AIC** | **BIC** | **saBIC** | **Entropy** |
| --- | --- | --- | --- | --- | --- | --- |
| **Female subsample** | | | | | |  |
| **1** | - | - | 17157.755 | 17210.001 | 17181.406 | - |
| **2** | 460.346 | **<**.001 | 16690.664 | 16766.130 | 16724.826 | .585 |
| **3** | **67.090** | **.0265** | **16629.425** | **16728.112** | **16674.099** | **.572** |
| **4** | 43.729 | .5046 | 16592.296 | 16714.202 | 16647.480 | .529 |
| **Male subsample** | | | | | |  |
| **1** | - | - | 15603.294 | 15655.395 | 15626.800 | - |
| **2** | **455.302** | **<.001** | **15141.378** | **15216.636** | **15175.332** | **.725** |
| **3** | 129.210 | .0715 | 15016.021 | 15114.434 | 15060.422 | .656 |

*Note.* Model selected based on LMR test shown in bold.

**Table S5: Model fits female and male subsamples for joint ADHD, internalizing, and conduct problem symptom trajectories**

| **N Classes** | **LMR** | ***p*** | **AIC** | **BIC** | **saBIC** | **Entropy** |
| --- | --- | --- | --- | --- | --- | --- |
| **Female subsample** | | | | | |  |
| **1** |  |  | 49520.051 | 49676.799 | 49591.014 |  |
| **2** | 2133.375 | <.001 | 47379.345 | 47594.147 | 47476.589 | .749 |
| **3** | **383.252** | **<.001** | **47011.183** | **47284.040** | **47134.710** | **.619** |
| **4** | 253.518 | .0836 | 46774.417 | 47105.329 | 46924.226 | .672 |
| **Male subsample** | | | | | |  |
| **1** |  |  | 47652.414 | 47808.751 | 47722.966 |  |
| **2** | 1760.084 | <.001 | 45889.736 | 46103.977 | 45986.419 | .682 |
| **3** | **430.477** | <.001 | **45473.733** | **45745.876** | **45596.546** | **.662** |
| **4** | 267.323 | .0739 | 45222.978 | 45553.024 | 45371.923 | .672 |

*Note.* Model selected based on LMR test shown in bold.

**Appendix S1. Trajectory group descriptions**

**ADHD symptoms**

In the female sub-sample, the first class (43.8% of the female sub-sample) was characterized by ADHD symptom levels that were intermediate between the symptom levels of the other two female classes. Their symptoms remained relatively constant between a score of 4 and 5, below the borderline and clinical thresholds. This class was thus labelled ‘mildly affected’. The second class (17.1%) showed the highest level of ADHD symptoms in the female sub-sample. Though scores began below the borderline and clinical cut-offs at age 10, from here they increased curvilinearly, plateauing in the clinical range by around age 13. This class was, therefore, labelled ‘high/adolescent increasing’. The third class in the female sub-sample (34.7%) had the lowest levels of ADHD symptoms overall, with SDQ scores constant around 2. This group was, therefore, labelled ‘unaffected’.

In the male subsample, the first class (17.5% of the male subsample), showed the highest level of ADHD symptoms overall. Symptoms began in the borderline range and increased further into the clinical range by around age 11. They remained in the clinical range until around age 15, with a peak between ages 12 and 13. This class was, therefore, labelled ‘high/adolescent-peaking’. The second class (39.4%) showed consistently low levels of ADHD symptoms (scoring between 2 and 3) and was, therefore, labelled ‘unaffected’. The third and largest class (43.1%) showed moderate and consistent symptom levels (around a score of 5) and was, therefore, labelled ‘mildly affected’. As compared to females, all of the male classes showed higher levels of ADHD symptoms compared with the most closely corresponding female class.

**Conduct problems symptoms**

In the female subsample, the first and largest class (76.5% of the subsample) was characterized by consistently and low levels of conduct problems. This class was thus labelled ‘unaffected’ The second class (23.3%) was characterized by consistently moderate levels, with only a very slight increase in levels in adolescence. This class was thus labelled ‘stable moderate’.

In the male subsample, the first and largest class (66.8%) was very similar to the female ‘unaffected’ class and was thus given the same label. The second class (6.1%) was characterized by initially high levels of conduct problems that further increased between early and middle adolescence. It was thus labelled ‘high increasing’. The third class (27.1%) was similar to the female ‘stable moderate’ class and was thus assigned this label too.

**Internalizing problems symptoms**

In the female subsample the first class (9.3% of the subsample), was characterized by initially high levels of internalizing problems that showed a curvilinear increase between early and middle adolescence, peaking around age 13-14. This class was labelled ‘high/adolescent peaking’. The second class (34.5%) was characterized by initially moderate levels that also increased between early and middle adolescence; however, levels remained lower than those in the first class across this period. This class was labelled ‘moderate increasing’. The third class (56.2%) was characterized by consistently low levels of internalizing problems and was thus labelled ‘unaffected’.

In the male subsample, the first class (18.9%) showed similar levels of and increases in internalizing problems to the female ‘moderate increasing’ group and was, therefore, also assigned this label. The second group (81.8%) showed low, slightly decreasing levels of internalizing problems between early and middle adolescence and was, therefore, labelled ‘low decreasing’.

**Joint trajectory groups**

In the female subsample, levels of ADHD symptoms, conduct problems, and internalizing problems tracked one another across the groups, with the highest, intermediate, and lowest levels of each occurring within the same groups as the highest, intermediate, and lowest levels of the others.

The first group (30.0%) of the sample was characterized by consistently low levels of symptoms in all three domains and was thus labelled ‘unaffected’. The second group (47.9%) of the sample was characterized by stable and slightly elevated levels in all three domains, especially ADHD symptoms, though these remained well below the clinical threshold. This group was labelled ‘mildly affected’. The third group (22.1%) was characterized by the highest levels of symptoms in all three domains and for ADHD, these symptoms were increasing over early to middle adolescence and crossed into the borderline range. Symptoms were also on an increasing trajectory for internalizing problems in this group. This group was labelled ‘high with increasing ADHD and internalizing problems’.

For the male subsample, the first class (12.6% of the sample) was characterized by high levels of ADHD and conduct problems symptoms and moderate levels of internalizing symptoms. The ADHD and conduct problems symptoms were the highest of any male or female class and for the former they crossed into the clinical range. The internalizing problems levels were similar to those observed in the ‘high with increasing ADHD and internalizing problems’ female class. Unlike this class; however, there was no increasing trajectory for any domain across early to middle adolescence. This group was thus labelled ‘stable high’. The second class (45.5%) was characterized by stably low levels in all three symptom domains and was thus labelled ‘unaffected’. Compared with the analogous female group, ADHD symptoms were higher and conduct problems were slightly lower. The third class (41.9%) was characterized by elevated levels of ADHD symptoms that sat just below the threshold for borderline symptoms. Conduct and internalizing problems were also elevated relative to the ‘low stable’ class but were not as high as in the ‘stable high’ class. There was no marked increasing or decreasing trend over early to middle adolescence. This class was thus labelled ‘moderate ADHD symptoms with mild conduct and internalizing problems’.
